# Baicalein, Ethyl Acetate, and Chloroform Extracts of *Scutellaria baicalensis* Inhibit the Neuraminidase Activity of Pandemic 2009 H1N1 and Seasonal Influenza A Viruses

**DOI:** 10.1155/2013/750803

**Published:** 2013-06-20

**Authors:** Mann-Jen Hour, Su-Hua Huang, Ching-Yao Chang, Yen-Kuan Lin, Ching-Ying Wang, Yuan-Shiun Chang, Cheng-Wen Lin

**Affiliations:** ^1^School of Pharmacy, China Medical University, No. 91 Hsueh-Shih Road, Taichung 40402, Taiwan; ^2^Department of Biotechnology, Asia University, No. 500 Lioufeng Road, Wufeng, Taichung 41354, Taiwan; ^3^Department of Medical Laboratory Science and Biotechnology, China Medical University, No. 91 Hsueh-Shih Road, Taichung 40402, Taiwan; ^4^School of Chinese Pharmaceutical Sciences and Chinese Medicine Resources, China Medical University, No. 91 Hsueh-Shih Road, Taichung 40402, Taiwan

## Abstract

This study rated antiviral activity of *Scutellaria baicalensis* Georgi (*S. baicalensis*) extracts against influenza A virus subtypes, for example, pandemic 2009 H1N1, seasonal H1N1 and H3N2. Ethyl acetate (EtOAc) and chloroform extracts inhibited *in vitro* neuraminidase (NA) enzymatic activity and viral replication more than methanol (MeOH) extract. EtOAc extract demonstrated NA inhibition IC_50_ values ranging from 73.16 to 487.40 *μ*g/mL and plaque reduction IC_50_ values ranging from 23.7 to 27.4 *μ*g/mL. Chloroform extract showed antiviral activities with plaque reduction IC_50_ values ranging from 14.16 to 41.49 *μ*g/mL Time-of-addition assay indicated that EtOAc and chloroform extracts also significantly inhibited virus yields after infection. HPLC analysis demonstrated that baicalin was dominant in the MeOH extract; baicalein and chrysin were rich in the EtOAc and chloroform extracts. Molecular simulation revealed baicalein hydrogen bonding with Glu277 as well as hydrophobic and Van der Waals interactions with Ile222, Arg224, Ser246, and Tyr347 in NA1 active sites of NA1. Baicalein inhibited in vitro replication of influenza A viruses pandemic 2009 H1N1 (IC_50_ = 0.018 *μ*M) and seasonal 2007 H1N1 using plaque reduction assays. A combination of low-dose baicalein with other anti-influenza agents could be applicable for development of alternative remedies treating influenza A virus infection.

## 1. Introduction

Influenza A virus, a member of the Orthomyxoviridae family, is an enveloped virus containing eight segmented, negative-sense, single-stranded RNAs [1, 2]. The viral genome encodes 10 proteins, for example, hemagglutinin (HA), neuraminidase (NA), M1, M2, nonstructural protein 1 (NS1), NP, and RNP. HA, NA, and M2 locate on the outer side of a viral envelope and M1 on the inner side. Of these envelope proteins, HA contains the receptor-binding site, being responsible for virus fusion and infectivity [3]. NA digests sialic acid on the cell surface, resulting in release of virus particles and spread of the virus. M2 ion channel modulates the acidic pH of the endosome, allowing the acidification of the internal virion core as well as causing release of vRNP into cell cytoplasm. Envelope proteins (HA, NA, and M2) play key roles in viral infectivity, making them prime targets for prophylaxis and therapeutic anti-influenza A virus drugs. Still, oseltamivir-resistant and amantadine-resistant variants emerge and are identified in several countries [4–9], creating a need to develop new anti-influenza compounds.

Influenza A virus consists of 17 HA and 10 NA subtypes [10]. Human influenza A viruses H1N1 and H3N2 subtypes commonly cause acute respiratory disease. Avian influenza viruses H5N1, H7N3, H7N7, and H9N2 subtypes occasionally infect humans [11]. A 2009 reassortment (pandemic 2009 H1N1) of avian, swine, and human influenza A viruses caused global outbreak, first human pandemic of its kind in the 21st century [12, 13]. Pandemic 2009 H1N1 rapidly spread worldwide, infecting 1 to 3 billion people from April 2009 to August 2010. Its virus replicates in mucosal epithelial cells of upper and lower airways, lung pneumocytes, alveolar macrophages, mucous glands, and lymph nodes, causing pathology similar to 1918 H1N1 and H5N1 viruses [14, 15]. Oseltamivir-resistant pandemic 2009 H1N1 isolates with H275Y mutation in NA were identified in 2010 [16]; drug-resistant pandemic 2009 H1N1 variants increased worldwide [17]. Importantly, a novel avian-origin influenza A (H7N9) virus causes an outbreak with severe and fatal respiratory diseases in China in 2013 [18], becoming global public health concerns, such that new therapies and vaccines against influenza infection become urgent.

*Scutellaria baicalensis *Georgi (“Huang-Qin” in Chinese) is a traditional Chinese medicinal herb exhibiting many biological activities, for example, antipyretic, antibacterial, antiviral, and/or anti-inflammatory properties [19]. Extracts of *S. baicalensis* inhibit growth of urothelial carcinoma cells [20], inducing apoptosis of human monocytic leukemia and osteogenic sarcoma cells [21]. *S. baicalensis* extracts modulate production of cytokines, linking with the antiviral activity [22]. Baicalin, baicalein, wogonin, wogonin 7-O-glucuronide, oroxylin A, oroxylin A 7-O-glucuronide, apigenin, and chrysin are major and bioactive components of *S. baicalensis* [23]. Baicalin and baicalein inhibit viral replication of parainfluenza [24], influenza A [25], hepatitis B [26], HIV-1 [27], and SARS coronavirus [28]. Wogonin inhibits hepatitis B surface antigen secretion while reducing HBV-DNA level *in vitro*, as confirmed by the animal model infected with duck hepatitis B virus [29]. Apigenin also inhibits *in vitro* replication of influenza and adenoviruses [30]. In our laboratory, *S. baicalensis* water extract shows inhibitory effects on *in vitro* enzymatic activity of influenza A virus NA. This study further probes antiviral activity of ethyl acetate (EtOAc), methanol (MeOH), and chloroform extracts against influenza A virus subtypes like pandemic 2009 H1N1 and seasonal influenza A viruses H1N1 and H3N2. In addition, molecular simulation and *in vitro* assays indicated flavonoids of S. *baicalensis*, such as baicalein and baicalin, as potentials of NA inhibitory agents.

## 2. Materials and Methods

### 2.1. Viruses and Cells

Pandemic influenza A/Taiwan/CMUH/2009 (H1N1) (pandemic 2009 H1N1 virus), seasonal influenza A/Taiwan//CMUH/2007 (H1N1), 2009 (H1N1), and 2009 (H3N2) (seasonal 2007 H1N1, 2009 H1N1, and 2009 H3N2 influenza A viruses) were isolated in the clinical virology laboratory of China Medical University Hospital, Taiwan. Influenza A/Puerto Rico/8/34 (H1N1) (PR8 H1N1 virus) was kindly provided by Dr. Wei-Li Hsu (Institute of Microbiology and Public Health, National Chung-Hsing University). HA subtype of all influenza A viruses used was confirmed by RT-PCR or real-time RT-PCR with specific primers (see Supplementary Table 1 in the Supplementary Material available online at http://dx.doi.org/10.1155/2013/750803).

 Madin-Darby canine kidney (MDCK) cells were maintained in Dulbecco's Modified Eagle's Medium (DMEM) supplemented with 10% fetal bovine serum, 100-fold dilution of penicillin-streptomycin solution (HyClone), and 250 *μ*g/mL amphotericin, as used for influenza A virus amplification and plaque assays.

### 2.2. *S. baicalensis* Extracts and Indicated Flavonoids

Thirty grams of *S. baicalensis* crude powder (Sun Ten Pharmaceutical Co., Ltd.) were dissolved in 200 mL ethyl acetate (EtOAc), methanol (MeOH), or chloroform and then gently sonicated 30 min at room temperature. Extract solutions centrifuged were filtered with Whatman No. 1 filter paper, then lyophilized by the freeze dryer (IWAKI FDR-50P). Each lyophilized extract powder was kept at −20°C; stock solutions (1 mg/mL) dissolved in phosphate-buffered saline and sterilized using a 0.44 *μ*m syringe filter were stored at −80°C until used. 

### 2.3. Fingerprint Analysis by HPLC

Baicalein, baicalin, chrysin, and apigenin that were purchased from Sigma Chemical Co. (St. Louis, MO, USA) were used as marker compounds of *S. baicalensis* flavonoids. Fingerprint profiles of *S. baicalensis* extracts were analyzed and compared with retention time of marker compounds, using HITACHI HPLC system (HITACHI, Japan) with quaternary pump (pump L-2130), a UV detector (L-2400), and a Waters XBridge C18 column (5 *μ*m, 4.6 × 100 mm, Waters). Mobile phase was performed as the linearly gradient from 100% acetonitrile to 0.5% sodium acetate in water during the period of 20 min. Chromatographic separation is set at 1.0 mL/min flow rate, elution peaks are detected at 280 nm, and each peak area is autocalculated with a 2996 PDA detector.

### 2.4. Enzymatic Assay of NA Activity by Fluorometric Substrate

Fluorometric substrate 2′-(4-methylumbelliferyl)-*α*-D-*N*-acetylneuraminic acid (MUNANA; Sigma) was used to determine NA activity as described in a prior report [31]. To determine NA activity of pandemic 2009 H1N1 and seasonal 2007 H1N1 viruses, serial 10-fold dilution of viruses (10^6^ PFU/mL) was added to the wells of a 96-well plate, mixed with MUNANA solution at final concentration of 300 *μ*M, and then incubated for 1 h at 37°C. Relative NA enzymatic activity was determined as the fluorescent intensity at a 360 nm excitation and a 460 nm emission wavelength (Multidetection Fluorescence-Luminescence Microplate Reader). For examining the inhibitory effects of *S. baicalensis* on NA activity, serial dilution of each extract or flavonoid was preincubated with each subtype of influenza A virus (10^5^ PFU/mL) for 1 h at 37°C, and mixture followed to react with MUNANA solution for another hour. Concentration of each extract or flavonoid showing 50% inhibitory effect (IC_50_) compared to the control with no inhibitor was determined by computer program (provided by John Spouge, National Institutes of Health).

### 2.5. Cytotoxic Assay for Extracts and Flavonoids of *S. baicalensis*

MDCK cells were cultured in 96-well plates, followed by 48 h incubation after adding medium containing each *S. baicalensis * extract (0, 100, 200, or 1000 *μ*g/mL), baicalein (0, 0.01, 0.1, 1, or 10 *μ*M), baicalin (0, 0.001, 0.01, 0.1, or 1 *μ*M), apigenin (0, 1, 10, 100, or 1000 *μ*M), or chrysin (0, 1, 10, 100, or 1000 *μ*M). Quintuplicate wells were performed for each concentration. Survival rates of cells were determined by MTT (3-(4,5-Dimethylthiazol-2-yl)-2,5-diphenyltetrazolium bromide) assay. Each well had 5 *μ*L of MTT solution (5 mg/mL) added for another 3 h, incubated, and washed three times with phosphate buffer saline before finally adding 100 *μ*L DMSO to wells for dissolving formazan crystals. Cell survival rates were calculated as ratio of optical density (OD_(570 nm)_ – OD_(630 nm)_) of treated cells to mock cells. Data represent mean ± SD of three independent experiments, and concentration giving 50% cytotoxic effect (CC_50_) was determined by ID50 computer program developed by Dr. John Spouge (National Institutes of Health).

### 2.6. Plaque Inhibition Assay for Anti-Influenza A Virus Activity of *S. baicalensis*

Confluent monolayers of MDCK cells in 6-well plates were inoculated with the influenza A virus (100 PFU) and immediately treated with/without *S. baicalensis* extract (0, 10, 100, or 1000 *μ*g/mL), baicalein (0, 0.001, 0.01, or 0.1 *μ*M), baicalin (0, 0.001, 0.01, or 0.1 *μ*M), apigenin (0, 10, 100, or 200 *μ*M), or chrysin (0, 10, 100, or 200 *μ*M). After 1 h absorption at 33°C, cells were overlaid with maintenance DMEM medium containing 1% agarose, 0.2% serum albumin, and 2.5 *μ*g/mL of trypsin. After 3 days of incubation at 33°C in a humidified atmosphere of 5% CO_2_, cells were stained with 0.1% crystal violet in 37% formaldehyde solution. Concentration giving 50% plaque inhibition (IC_50_) was determined, using ID50 computer program.

### 2.7. Cytopathic Effect, Virus Yield, and Time-of-Addition Assays

*S. baicalensis* extract (0, 10, 100, or 1000 *μ*g/mL), baicalein (0, 0.001, 0.01, or 0.1 *μ*M), baicalin (0, 0.001, 0.01, or 0.1 *μ*M), apigenin (0, 10, 100, or 200 *μ*M), or chrysin (0, 10, 100, or 200 *μ*M) was added to MDCK cells cultured in 6-well plates during (simultaneous treatment) and 1 h after (postinfection treatment) infection with pandemic 2009 H1N1 at MOI 1. Thirty-six h after infection, virus-induced cytopathic effect in each well was photographed using reverse-phase light microscopy; viral RNA genome in each cultured supernatant was extracted by QIAamp Viral RNA Mini Kit (Qiagen). Real-time RT-PCR was performed with specific primers for pandemic 2009 H1N1 (Supplemental Table 1), SYBR green PCR Master Mix, and SYBR Green I dsDNA binding dye by ABI PRISM 7000 sequence detection system (Applied Biosystems). Δ*C*_*t*_ value as relative viral RNA load was calculated by subtracting *C*_*t*_ value for viral load in cultured media of treated infected cells from *C*_*t*_ value in those of mock-infected cells. Δ*C*_*t*_ value above 3.3 indicated more than 1-log reduction (equal to 90% inhibitory concentration (IC_90_)) in virus RNA load.

### 2.8. Molecular Docking

 The crystal structures of neuraminidase NA1 (PDB: 3cl0), NA2 (PDB: 4gzp), and NA9 (PDB: 3nn9) deposited in the RCSB Protein Data Bank (http://www.rcsb.org/pdb) were used as the targets for molecular docking. The docking calculations of *S. baicalensis* flavonoids and Tamiflu with NA1, NA2, and NA9 were performed with LigandFit program within the software package Discovery Studio 2.5 (Accelrys, San Diego, USA), which is an automated tool for ligand-protein docking and scoring. The prepared protein protocol was used to NA structures including the following actions: standardize atom names, insert missing atoms in residues and remove alternate conformations, insert missing loop regions based on SEQRES data, optimize short and medium size loop regions with Looper algorithm, minimize remaining loop regions, and calculate p*K* and protonate structure.

## 3. Results

### 3.1. Inhibition of NA Activity by *S. baicalensis* Extracts

**Figure 1:**
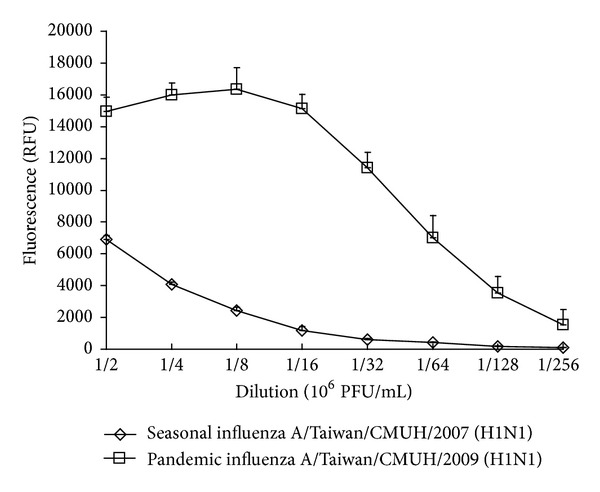
*In vitro* NA enzymatic activity of pandemic 2009 H1N1 and seasonal 2007 H1N1 influenza A viruses. Fluorometric substrate MUNANA (300 *μ*M) was incubated with serial 10-fold dilution of viruses (10^6^ PFU/mL) and then incubated for 1 h at 37°C. Relative NA enzymatic activity was determined as the fluorescent intensity at a 360 nm excitation and a 460 nm emission wavelength.

To screen inhibitory effects of *S. baicalensis* extracts on NA enzymatic activity, fluorometric activity assay of NA with MUNANA substrate indicated NA enzymatic activity of pandemic 2009 and seasonal 2007 H1N1 influenza A viruses by virus titer-dependent manner (Figure 1). Meanwhile, pandemic 2009 H1N1 influenza A virus exhibited greater NA activity than seasonal 2007 H1N1 influenza A virus. Subsequently, MeOH, EtOAc, and chloroform extracts of *S. baicalensis* were prepared to test their inhibitory effects on NA activity of five variants: pandemic 2009 H1N1, seasonal 2007 H1N1, 2009 H1N1, 2009 H3N2, and PR8 H1N1 influenza A viruses (Table 1). EtOAc and chloroform extract inhibited NA enzymatic activity of these variants more potently than MeOH extract. Ranking IC_50_ value of EtOAc extract on inhibiting NA activity of the variants from lowest to highest saw seasonal 2007 H1N1 (73.16 *μ*g/mL), 2009 H1N1 (176.57 *μ*g/mL), 2009 H3N2 (306.96 *μ*g/mL), pandemic 2009 H1N1 (388.98 *μ*g/mL), and PR8 H1N1 influenza A viruses (487.40 *μ*g/mL) (Table 1). Therefore, *S. baicalensis* extracts as NA inhibitors further examined inhibitory effect on replication of influenza A viruses.

### 3.2. Inhibition of Influenza A Virus Replication by *S. baicalensis* Extracts

Cytotoxicity of* S. baicalensis* extract to MDCK cells was examined by MTT assay (Table 2); these extracts proved less toxic (CC_50_ ≥ 800 *μ*g/mL) as available for *in vitro* activity against influenza A viruses. Plaque inhibition assay indicated *S. baicalensis* extracts concentration dependently inhibiting replication of pandemic 2009 H1N1 and seasonal 2007 H1N1 influenza A viruses (Table 2). IC_50_ values against seasonal 2007 H1N1 influenza A virus were 23.7 *μ*g/mL for EtOAc extract and 41.5 *μ*g/mL for chloroform extract; IC_50_ values against pandemic 2009 H1N1 influenza A virus were 27.4 *μ*g/mL for EtOAc extract and 14.2 *μ*g/mL for chloroform extract, respectively, and therapeutic indexes above 30 against both pandemic 2009 H1N1 and seasonal 2007 H1N1 influenza A viruses.

### 3.3. Inhibition of Pandemic 2009 H1N1 Influenza A Virus Yield by Time-of-Addition with *S. baicalensis* Extracts

**Figure 2:**
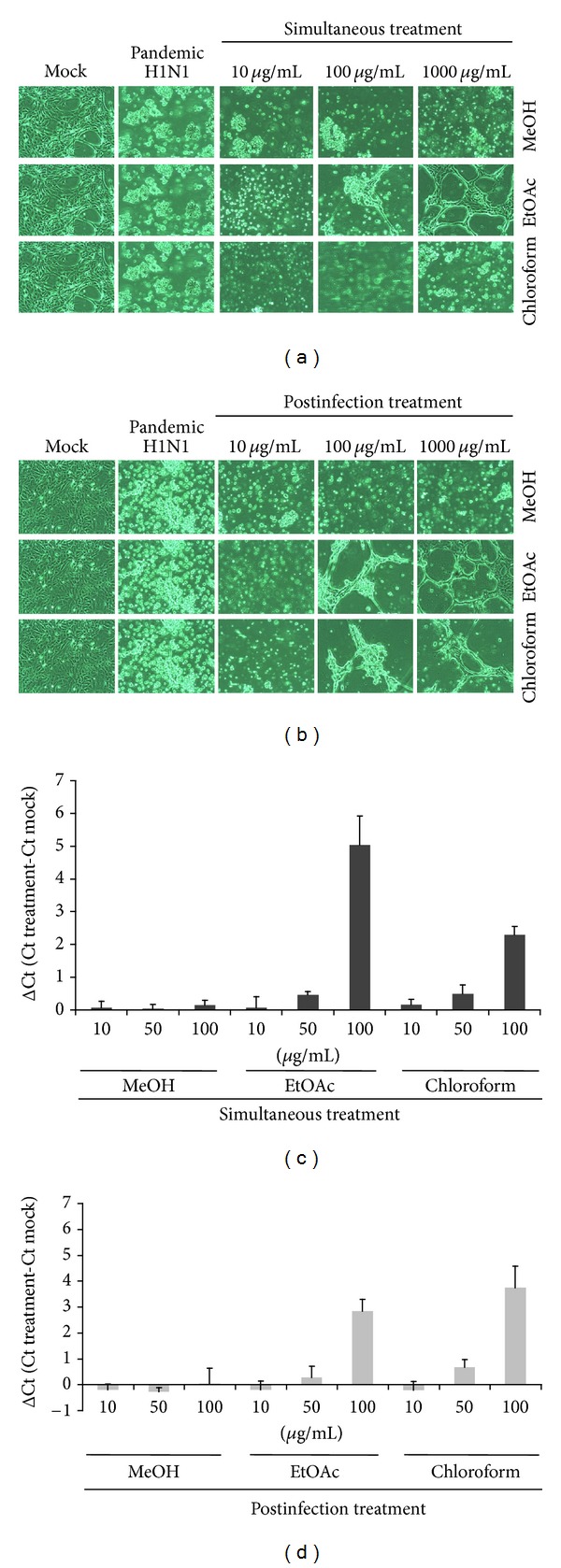
Time-of-addition inhibition of *S. baicalensis* extracts on pandemic 2009 H1N1 influenza A virus yields. Indicated extracts (0, 10, 100, or 1000 *μ*g/mL) treated MDCK cells during (simultaneous treatment) (a) and 1 h after (postinfection treatment) (b) infections of pandemic 2009 H1N1 virus. After 36 h incubation, virus-induced cytopathic effect was photographed, using reverse-phase light microscopy. (c and d) Virus yield was determined real-time RT-PCR; Δ*C*_*t*_ value was calculated by subtracting *C*_*t*_ value for viral load in cultured media of treated infected cells from *C*_*t*_ value in those of mock-infected cells.

To examine time-of-addition effect on virus yield, MDCK cells were treated simultaneously (at the same time as infection) or after infection (after entry) with various concentrations of *S. baicalensis* extracts. EtOAc and chloroform extracts, but not MeOH extract, showing concentration-dependent inhibition of cytopathic effect as well as virus yield with simultaneous and postinfection treatment (Figure 2). Real-time RT-PCR assay indicated simultaneous and postinfection treatment of EtOAc and chloroform extract (100 *μ*g/mL) causing more than 1-log reduction in virus RNA loads (Δ*C*_*t*_ value greater than 3.3) compared to mock-infected supernatant.

### 3.4. HPLC Analysis of Flavonoids in *S. baicalensis* Extracts

**Figure 3:**
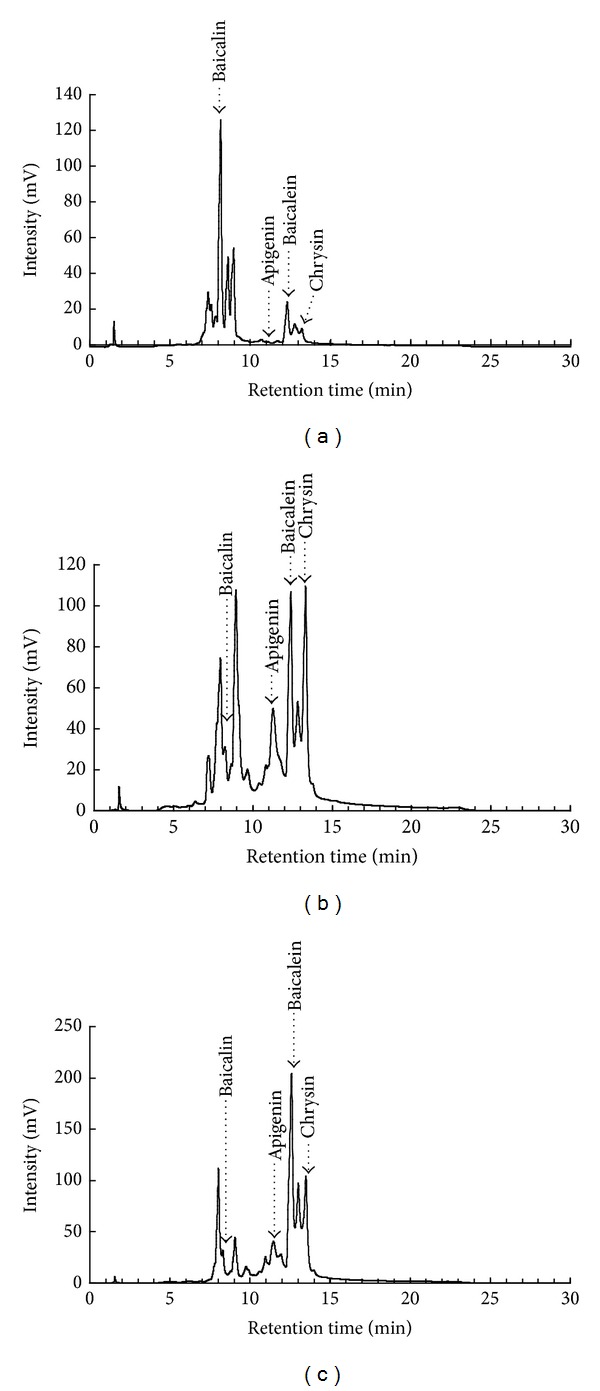
HPLC fingerprint profiles of *S. baicalensis* extracts. Marker components (baicalein, baicalin, apigenin, and chrysin), as well as the MeOH (a), EtOAc (b), and chloroform (c) extracts, were analyzed by HPLC with C-18 reverse phase column, and eluents are detected at 280 nm with a 2996 PDA detector.

**Figure 4:**
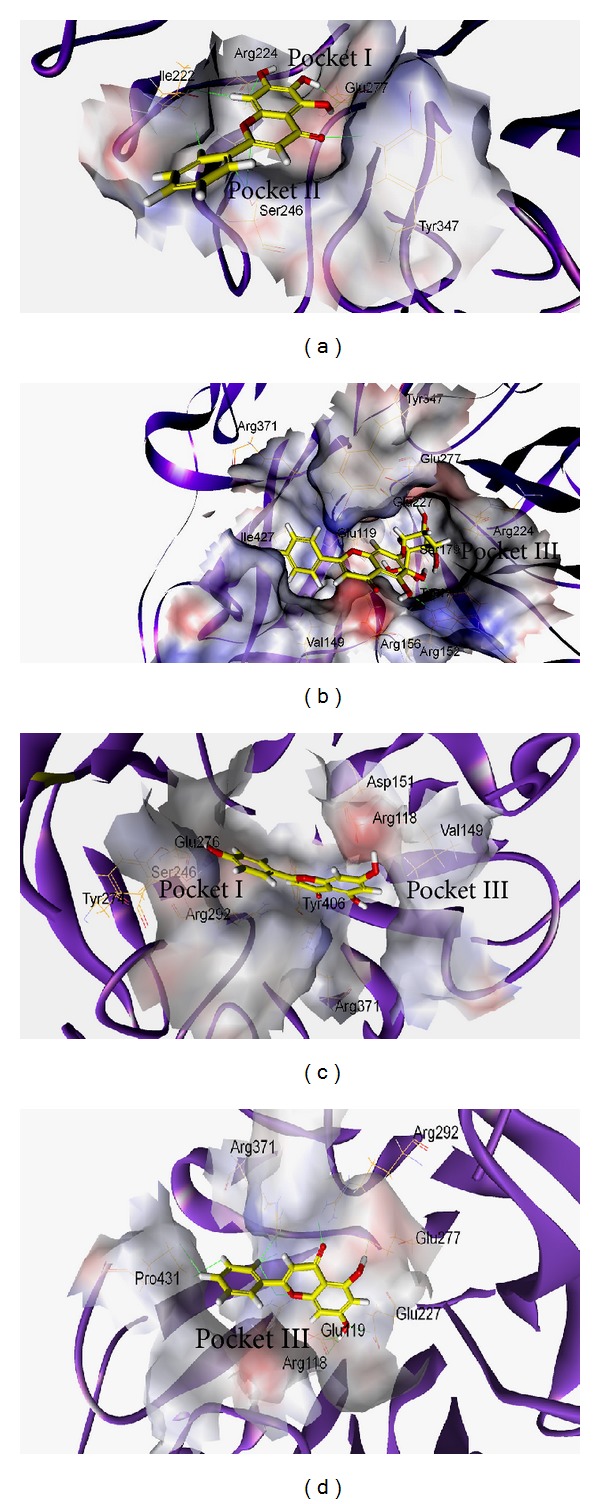
Molecular modeling. Baicalein (a), baicalin (b), apigenin (c), and chrysin (d) docked well with NA1 active sites. The binding amino acids are shown as lines and labels. The carved surface representation of the pocket formed from flavonoid binding is shown as transparent gray.

*S. baicalensis* contains more than 200 compounds identified with over 40 flavonoids. Six major bioactive flavonoids in *S. baicalensis* are baicalein, baicalin, wogonin, wogonside, oroxylin, and oroxylin A-7-glucuronide; minor bioactive flavonoids have chrysin, chrysin-6,8-di-C-glucoside, apigenin, apigenin-6-C-glucose-8-C-arabinose, and so forth [23]. To examine fingerprint of *S. baicalensis* extracts, baicalein, baicalin, apigenin, and chrysin were used as standard marker components; these three extracts were analyzed using HPLC with C-18 reverse phase column (Figure 3). The retention time of HLPC chromatograph at 280 nm was at 8.20 min for baicalin, 11.10 min for apigenin, 12.40 min for baicalein, and 13.10 min for chrysin, respectively. HPLC chromatogram indicated that the concentrations of baicalin, apigenin, baicalein, and chrysin were 29.68%, 0.09%, 7.48%, and 4.44% in the MeOH extract (Figure 3(a)), 3.30%, 14.23%, 14.04%, and 17.76% in the EtOAc extract (Figure 3(b)), and 0.47%, 0.74%, 25.30%, and 18.30% in the chloroform extract (Figure 3(c)), respectively. In addition to these four makers used, HPLC chromatogram demonstrated other components in each extract. For analyzing the association of relative concentrations of flavonoids with antiviral activities of different extracts, baicalein, baicalin, apigenin, and chrysin against influenza A viruses were further rated by molecular docking with NA, NA enzymatic inhibition, and plaque reduction assays (Tables 3–6, Figure 4).

### 3.5. Molecular Interaction of *S. baicalensis* Flavonoids with NA1, NA2, and NA9

In order to predict the vital antiviral components of *S. baicalensis* EtOAc and chloroform extracts, the computational simulation of *S. baicalensis* flavonoids such as baicalein, baicalin, apigenin, and chrysin, with influenza A viruses NA1, NA2, and NA9, was performed. We scored our models using two scoring functions, LigScore and DockScore, and in addition, Tamiflu was used as a positive control in this docking experiment. The results showed that the *S. baicalensis* flavonoids, especially baicalin and baicalein, bond well to NA1, NA2, and NA9, with the high affinity based on higher score values using LigScore2_Dreiding and DockScore programs (Table 4). As shown in Table 4 and Figure 4(a), baicalein interacted with Ile222, Arg224, Ser246, Glu277, and Tyr347 in Pocket I and II of NA1 (PDB: 3cl0) via hydrogen bonding and Van der Waals interactions. Interaction of baicalin with NA1 showed five hydrogen bonds between baicalin and Glu119, Arg152, Arg156, and Glu277 as well as Van der Waals interactions between the ligand and Glu119, Val149, Arg152, Arg156, Trp178, Ser179, Arg224, Glu227, Arg371, Tyr347, and Ile427 in Pocket I and III of NA1 (Figure 4(b) and Table 4). As to the simulation of apigenin or chrysin with NA1 (Figures 4(c) and 4(d)), hydrogen bonds and Van der Waals interactions were present between the ligand and the residues in Pocket I and III of NA1. These interactions showed baicalein directly interacting with the hydrophobic pocket (Pocket II) formed by highly conserved residues of NA, but baicalin, apigenin, or chrysin hydrogen bonding with the charge residues in Pocket III of NA1. The results indicated these four flavonoids directly interacting with NA1 active-site residues; baicalein showed the unique interaction with NA1, particularly via hydrophobic interactions with Ile222, Arg224, and Ser246.

### 3.6. NA Inhibition and Plaque Reduction by *S. baicalensis* Flavonoids

**Figure 5:**
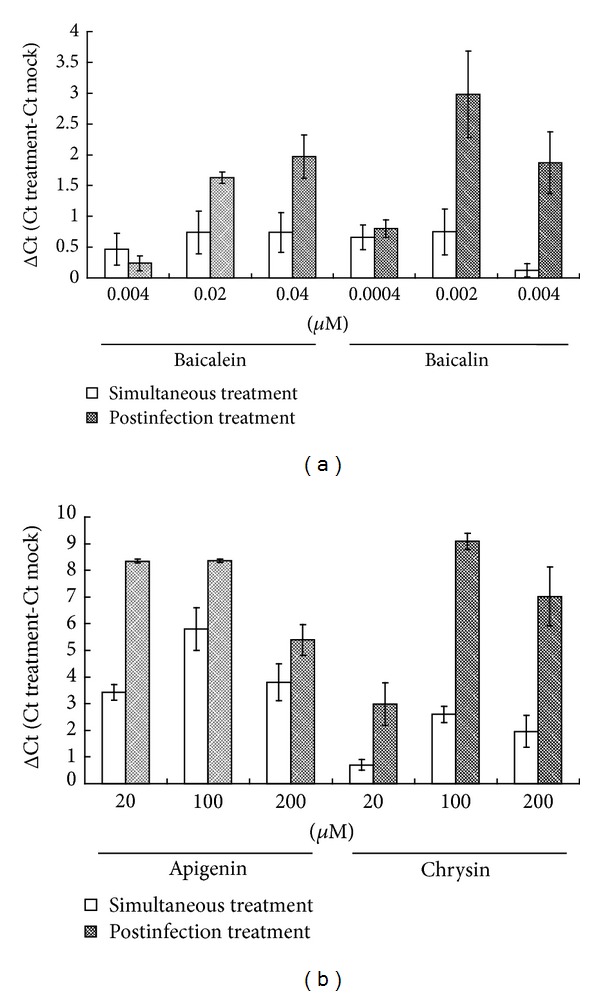
Time-of-addition inhibition of *S. baicalensis* flavonoids on pandemic 2009 H1N1 virus yields. Baicalein, baicalin (a), apigenin, or chrysin (b) was incubated with MDCK cells during (simultaneous treatment) and 1 h after (postinfection treatment) infections of pandemic 2009 H1N1 influenza A virus. Virus yield was determined real-time RT-PCR; Δ*C*_*t*_ value was calculated by subtracting *C*_*t*_ value for viral load in cultured media of treated infected cells from *C*_*t*_ value in those of mock-infected cells.

Flavonoids baicalein, baicalin, apigenin, and chrysin were further tested for inhibitory ability of NA enzymatic activity, using fluorometric assay (Table 5). IC_50_ NA inhibition values against five variants were 0.18~0.53 *μ*M for baicalein, 2.55~5.84 *μ*M for baicalin, 61.7~118.48 *μ*M for apigenin, and 109.64~465.11 *μ*M for chrysin, respectively. In plaque reduction assay, these flavonoids exhibited concentration-dependent inhibitory effect. In particular, IC_50_ plaque reduction values of baicalein were 0.018 *μ*M against seasonal 2007 H1N1 influenza A virus and 0.02 *μ*M against pandemic 2009 H1N1 influenza A virus (Table 6). Of them, only baicalein showed therapeutic index greater than 2 (CC_50_/IC_50_ plaque reduction) against both variants. Interestingly, virus yield assay with real-time PCR indicated these flavonoids with higher antiviral activity after infection than simultaneous treatment (Figure 5).

## 4. Discussion

This study demonstrated different NA enzymatic activity of influenza A subtypes as well as high NA activity of pandemic 2009 H1N1 influenza A virus and low NA activity of seasonal 2007 H1N1 influenza A virus (Figure 1), correlating with *in vitro* NA sensitivity to *S. baicalensis* extracts (Table 1). IC_50_ values against pandemic 2009 H1N1 influenza A virus by EtOAc (388.98 *μ*g/mL) and chloroform (562.94 *μ*g/mL) extracts were higher than IC_50_ values against seasonal 2007 H1N1 influenza A virus (73.17 and 109.71 *μ*g/mL, resp.); EtOAc and chloroform extracts showed higher NA inhibitory ability with lower IC_50_ values against these variants compared to MeOH extract. Interestingly, the plaque reduction assay indicated EtOAc and chloroform extracts as lower IC_50_ plaque inhibition values than IC_50_ NA inhibition values against pandemic 2009 H1N1 and seasonal 2007 H1N1 influenza A viruses (Table 2). Time-of-addition assay revealed EtOAc and chloroform extracts reducing virus yield significantly in both simultaneous and postinfection treatment assays (Figure 2), also attesting to therapeutic potential of EtOAc and chloroform extracts against influenza A. These revealed EtOAc and chloroform extracts with multiple anti-influenza A virus actions, except for NA inhibition.

Over 30 flavonoids were identified from *S. baicalensis*, being linked with the antiviral activity of EtOAc and chloroform extracts. As shown in Figure 3, the retention time order of these four flavonoids in reverse phase C18 column was chrysin (13.10 min) > baicalein (12.40 min) > apigenin (11.10 min) > baicalin (8.20 min), being in accordance with the prior study [32]. Meanwhile, glycoside forms like baicalin, wogonside, and roxylin A-7-glucuronide of flavonoids in *S. baicalensis* were rich in the methanol extract, while aglycone forms such as baicalein and wogonin were identified in the ethyl acetate extract [33]. Both baicalein and chrysin were rich in EtOAc and chloroform extracts, being involved in antiviral actions of these two extracts against influenza A viruses. *In vitro* antiviral assays indicated baicalein as a potent NA inhibitor with NA inhibition IC_50_ less than 0.5 *μ*M (Table 5), significantly inhibiting the replication of influenza A viruses in cell cultures (plaque reduction IC_50_ less than 0.05 *μ*M) (Table 6).

Molecular modeling was initially used to predict the interaction of NA with *S. baicalensis* flavonoids, implying the correlation with the inhibitory activity of *S. baicalensis* extracts. Using LigScore2_Dreiding and DockScore elucidated molecular interactions between flavonoids and NA active sites such as hydrogen bonding, Van der Waals, lipophilic, and polar attractive/repulsive interactions. Ranking the docking scores of flavonoids by LigScore2_Dreiding and DockScore was baicalin > baicalein > apigenin > chrysin (Table 3). Table 4 and Figure 4 revealed, these four flavonoids binding with NA active sites, consisting of catalytic sites (Arg118, Asp151, Arg152, Arg224, Glu276, Arg292, Arg371, and Tyr406 in N2 numbering) and substrate binding and framework sites (Glu119, Arg156, Trp178, Ser179, Asp/Asn198, Ile222, Glu227, His274, Glu277, Asn294, and Glu425) [34]. Molecular modeling of baicalin/NA1 complex showed five hydrogen bonds between the ligand and Glu119, Arg152, Arg156, and Glu277 as well as Van der Waals interactions between the ligand and Glu119, Val149, Arg152, Arg156, Trp178, Ser179, Arg224, Glu227, Arg371, Tyr347, and Ile427. Meanwhile, molecular interaction between baicalein and NA1 had one hydrogen bond between the ligand and Glu277 as well as hydrophobic and Van der Waals interactions between the ligand and Ile222, Arg224, Ser246, Glu277, and Tyr347. The difference in the interactions of baicalein and baicalin with NA1 could correlate with more hydrophilic characters of baicalin with three hydroxyl groups and D-glucopyranosiduronic acid compared to baicalein. The reason could be responsible for the correlations between docking scoring and the antiviral activities of these four flavonoids in NA enzymatic inhibition and plaque reduction assays (Tables 5 and 6), in which the order of inhibitory efficacy and antiviral activity was baicalein > baicalin > apigenin > chrysin. The results suggested that the hydrophobic interaction of potential inhibitors with the highly conserved Pocket II (Ile222, Arg224, and Ser246) of NA1 provides the alternative approach to treat the NA inhibitor-resistant mutants such as His274Tyr, Glu119Val, and Arg292Lys mutation in NA1, NA2, and NA9. 

Of four *S. baicalensis* associated flavonoids used in this study, baicalein showed potent anti-influenza A virus activities with IC_50_ NA inhibition values ranging from 0.181 to 0.526 *μ*M and IC_50_ plaque reduction values ranging from 0.018 to 0.020 *μ*M (Tables 5 and 6). Baicalin also had potent anti-influenza A virus activities with IC_50_ ranging from 2.55 to 5.84 *μ*M. Apigenin and chrysin displayed moderate inhibitory effects against influenza A variants. In time-of-addition assay, postinfection treatment with these flavonoids had more potent inhibitory effect on virus yield compared to simultaneous treatment, linking with their NA inhibition activity and reduction of virus release into cultured supernatant. Baicalein exhibited a broad spectrum of antiviral activities, for example, dengue [35], influenza A H5N1 [36], Sendai [24], and human cytomegalovirus [37]. Baicalein inhibited NA activity of influenza A H5N1 and Sendai viruses [24, 36], echoing our finding NA inhibition by baicalein on pandemic 2009 H1N1 influenza A virus plus another four variants. Likewise, baicalein suppressed IL-6 and IL-8 production in H5N1-infected human monocyte-derived macrophages [36]. Aside from baicalein, baicalin manifested antiviral activity against influenza A/FM1/1/47 (H1N1) [25], HIV-1 [38], SARS coronavirus [26], and herpes simplex virus type 1 [39]. Baicalein inhibited Env protein-mediated fusion with chemokine receptors and CD4 during HIV-1 entry process [38]. Although the literature survey and our results indicated baicalein and baicalin exhibiting the potent anti-influenza and anti-inflammatory activities, both had low therapeutic index. A combination of baicalein with ribavirin demonstrated synergistic effects on inhibiting *in vitro* and *in vivo* replication of influenza A virus [40]. A combined treatment of baicalein/baicalin with other active agents could reduce cytotoxicity with lowering dosage of baicalein/baicalin and prove the anti-influenza potency.

*S. baicalensis* extracts contain many flavonoids, exhibiting a broad spectrum of antiviral activities, but processing different molecular mechanisms against viral infections. Except for these four flavonoids tested in this study, 5,7,4′-trihydroxy-8-methoxyflavone and 5,7,8,4′-tetrahydroxyflavone have been identified as *S. baicalensis*-associated flavonoids, exhibiting potent anti-influenza efficacy via inhibiting NA enzymatic activity [41–43]. *S. baicalensis* extracts suppressed HBV core gene promoter activity and led to inhibited virus production *in vitro* [44]. *S. baicalensis* extracts also modulated cytokine production of human peripheral blood leukocytes and then enhanced resistance of host cells to vesicular stomatitis virus infection [22]. Baicalein and wogonin reduced inflammation via suppressing cyclooxygenase-2 activity [45]. Regulation of inflammation, transcriptional activity, and cytokine production could associate with anti-influenza A virus activity of *S. baicalensis* extracts; we will further investigate in detail.

*S. baicalensis* EtOAc and chloroform extracts containing a high concentration of baicalein significantly inhibited *in vitro* NA activity and replication of influenza A virus subtypes, including pandemic 2009 H1N1 as well as seasonal H1N1 and H3N2 influenza A viruses. Comparison of therapeutic index among three extract types indicated EtOAc and chloroform extracts as potential therapeutic agents against influenza A virus. Among the flavonoids, baicalein, the key antiviral component in EtOAc and chloroform extracts, was an NA- specific inhibitor, showing potent anti-influenza A virus activity, yet highly cytotoxic to MDCK cells. Combining low-dose baicalein with other antiviral agents could be alternative remedies against influenza A virus infection.

## Supplementary Material

Virus subtyping and viral load by real-time RT-PCR: Viral RNA was extracted from culture supernatant using QIAamp Virus RNA Mini Kit (Qiagen). Viral RNA genome was detected a QIAGEN One-Step RT-PCR kit with the specific primer sets listed in Supplemental Table S1. The primer pairs were the following: H1F and H1R for seasonal influenza A H1, H3F and H3R for seasonal influenza A H3, and Sw-HA-F and Sw-HA-R for the pandemic 2009 influenza A virus. RT-PCR products were analyzed by agarose electrophoresis. For viral load assay, viral RNA was analyzed with real-time one-step RT-PCR assays with SYBR green PCR Master Mix, and SYBR Green I dsDNA binding dye. The specific primer pairs were M-F and M-R for influenza A virus, as well as Swl-M-F and Swl-M-R for the pandemic 2009 influenza A virus.

## Figures and Tables

**Table 1:** Inhibitory effects of MeOH, EtOAc, and chloroform extracts of *S. baicalensis* on NA enzymatic activity.

Extract	NA inhibition IC_50_ values (*μ*g/mL)				
	Seasonal 2007 H1N1	Pandemic 2009 H1N1	PR8 H1N1	Seasonal 2009 H1N1	Seasonal 2009 H3N2
MeOH	613.94 ± 5.54	>1000	>1000	875.92 ± 1.01	783.31 ± 0.23
EtOAc	73.17 ± 1.65	388.98 ± 0.93	487.40 ± 1.68	176.57 ± 0.32	306.96 ± 0.40
Chloroform	109.71 ± 3.08	562.94 ± 0.78	569.20 ± 0.52	251.29 ± 2.30	427.55 ± 0.59

**Table 2:** Plaque reduction of influenza A virus by MeOH, EtOAc, and chloroform extracts of *S. baicalensis*.

Extracts	CC_50_ (*μ*g/mL)	Plaque inhibition IC_50_ values (*μ*g/mL)		Therapeutic index	
	MDCK cells	Seasonal 2007 H1N1	Pandemic 2009 H1N1	Seasonal 2007 H1N1	Pandemic 2009 H1N1
MeOH	923.12 ± 0.017	134.22 ± 0.059	28.24 ± 0.049	6.9	32.7
EtOAc	836.24 ± 0.023	23.70 ± 0.053	27.39 ± 0.049	35.3	30.5
Chloroform	829.77 ± 0.009	41.49 ± 0.052	14.16 ± 0.083	20.0	58.6

**Table 3:** Molecular docking of *S. baicalensis* flavonoids into influenza A viruses NA1, NA2, and NA9.

Flavonoids	LigScore2_Dreiding			DockScore		
	NA1^a^	NA2^b^	NA9^c^	NA1^a^	NA2^b^	NA9^c^
Baicalein	4.42	3.76	4.68	61.041	49.358	36.683
Baicalin	6.52	4.13	5.24	77.712	48.391	38.279
Apigenin	4.51	3.66	4.41	57.727	44.794	35.018
Chrysin	4.92	3.64	4.37	54.621	40.560	34.109
Tamiflu	5.67	3.67	4.16	54.409	33.117	33.935

^a^Avian influenza virus A/Vietnam/1203/04 (H5N1).

^b^Influenza virus A/Tanzania/205/2010 (H3N2).

^c^Avian influenza virus A/tern/Australia/G70c/75 (N9).

**Table 4:** Key interactions of *S. baicalensis* flavonoids with NA1 active sites.

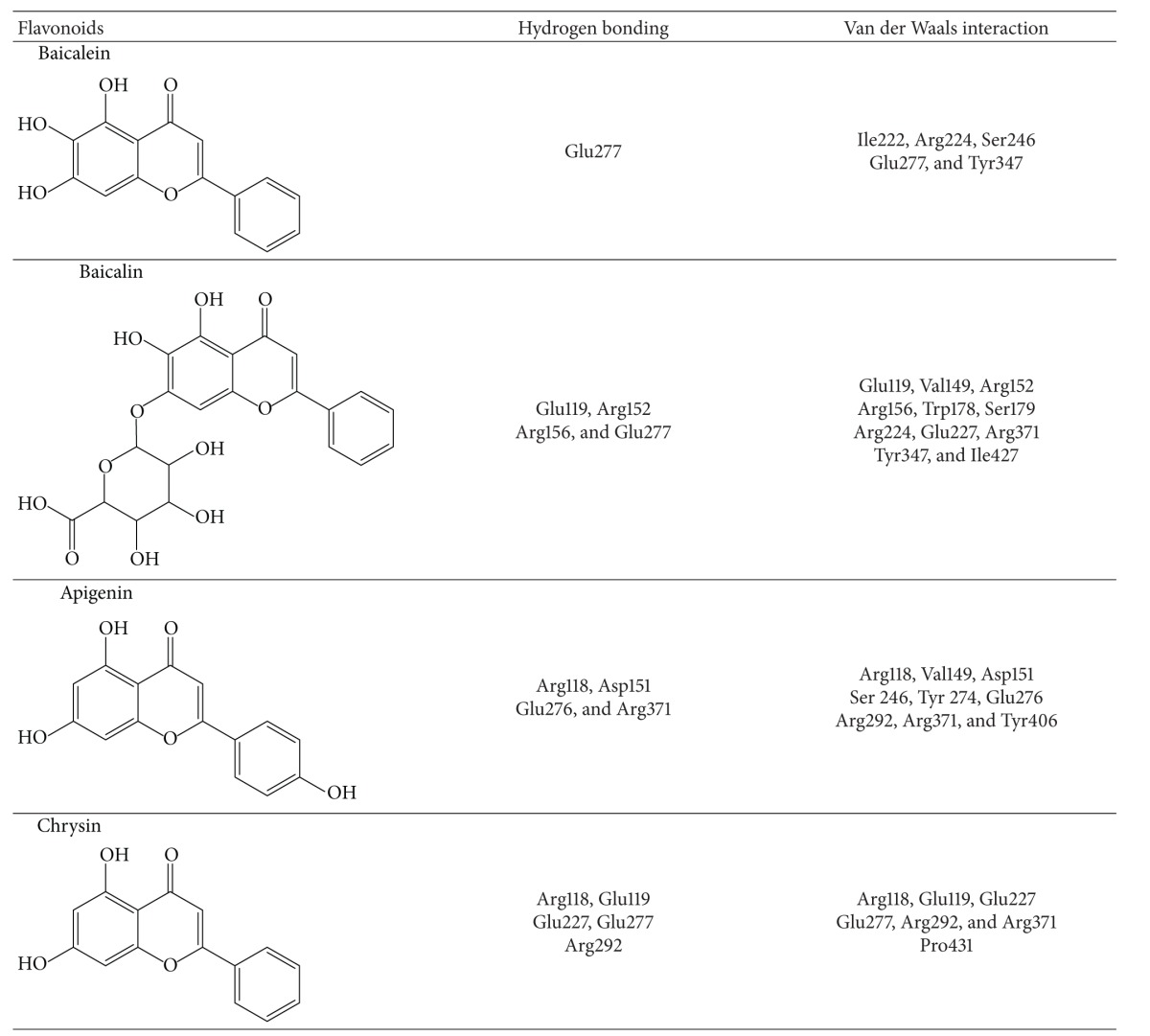

**Table 5:** Inhibitory effects of flavonoids of *S. baicalensis* on NA enzymatic activity.

Flavonoids	NA inhibition IC_50_ values (*μ*M)				
	Seasonal 2007 H1N1	Pandemic 2009 H1N1	PR8 H1N1	Seasonal 2009 H1N1	Seasonal 2009 H3N2
Baicalein	0.423 ± 0.069	0.287 ± 0.074	0.181	0.526 ± 0.084	0.436
Baicalin	2.55 ± 0.083	2.57 ± 0.077	2.754	5.84 ± 0.074	3.98
Apigenin	61.72	112.23	81.3	118.48 ± 0.06	83
Chrysin	109.64	465.11 ± 0.09	131.92	175.43 ± 0.70	194.93

**Table 6:** Plaque reduction of influenza A virus by flavonoids of *S. baicalensis*.

Flavonoids	CC_50_ (*μ*M)	Plaque inhibition IC_50_ values (*μ*M)		Therapeutic index	
	MDCK cells	Seasonal 2007 H1N1	Pandemic 2009 H1N1	Seasonal 2007 H1N1	Pandemic 2009 H1N1
Baicalein	0.045 ± 0.088	0.018 ± 0.062	0.020 ± 0.077	2.5	2.3
Baicalin	0.015	>0.01	>0.01		
Apigenin	218.34 ± 0.11	119.61 ± 0.032	94.16 ± 0.047	1.8	2.3
Chrysin	266.66 ± 0.077	>200	>200		
