# Supplementary material for: Baicalein, Ethyl Acetate, and Chloroform Extracts of Scutellaria baicalensis Inhibit the Neuraminidase Activity of Pandemic 2009 H1N1 and Seasonal Influenza A Viruses
Source: Evid Based Complement Alternat Med. 2013 Jun 20;2013:750803. doi: 10.1155/2013/750803 (PMC3705751; doi:10.1155/2013/750803)
Supplement: Supplementary file 1 — Virus subtyping and viral load by real-time RT-PCR: Viral RNA was extracted from culture supernatant using QIAamp Virus RNA Mini Kit (Qiagen). Viral RNA genome was detected a QIAGEN One-Step RT-PCR kit with the specific primer sets listed in Supplemental Table S1. The primer pairs were the following: H1F and H1R for seasonal influenza A H1, H3F and H3R for seasonal influenza A H3, and Sw-HA-F and Sw-HA-R for the pandemic 2009 influenza A virus. RT-PCR products were analyzed by agarose electrophoresis. For viral load assay, viral RNA was analyzed with real-time one-step RT-PCR assays with SYBR green PCR Master Mix, and SYBR Green I dsDNA binding dye. The specific primer pairs were M-F and M-R for influenza A virus, as well as Swl-M-F and Swl-M-R for the pandemic 2009 influenza A virus. [file 750803.f1.docx]

Supplemental Table 1. Primers for subtyping and viral load of influenza A viruses

| **Primer name** | | **Primer sequence** |
| --- | --- | --- |
| Influenza A virus M-F | | AAGACCAATCCTGTCACCTCTGA |
| Influenza A virus M-R | | CAAAGCGTCTACGCTGCAGTCC |
| Influenza A virus H1-F | | GATGCAGACACAATATGTAGAGG |
| Influenza A virus H1-R | | CNCTACAGAGACATAAGCATTT |
| Influenza A virus H3-F | | TCAGATTGAAGTGACTAATGCT |
| Influenza A virus H3-R | AATTTTGATGCCTGAAACCGT | |
| Influenza A virus Swl-M-F | TGTGCCACTTGTGAACAGATTG | |
| Influenza A virus Swl-M-R | CTGATTAGTGGATTGGTGGTAGTAGC | |
| Influenza A virus Sw-HA-F | TCAGTGTCATCATTTGAAAGGTTTG | |
| Influenza A virus Sw-HA-R | TTTTTGTAGAAGCTTTTTGCTCCAG | |
